# Second-Phase Grain Boundary Engineering in Al_2_O_3_-MgAl_2_O_4_ Composites: Microstructure and Dielectric Breakdown Reliability

**DOI:** 10.3390/ma19153263

**Published:** 2026-08-02

**Authors:** Yaling Yu, Wei Xu, Chenyang Zhang, Huan Yang, Shaomin Lin

**Affiliations:** 1 Advanced Ceramic Materials Innovation Research Center, Hanshan Normal University, Chaozhou 521041, China; 2 School of Materials Science and Engineering, South China University of Technology, Guangzhou 510640, China

**Keywords:** alumina, magnesium aluminate spinel, grain boundary engineering, dielectric breakdown, Weibull statistics

## Abstract

**Highlights:**

- A dual-pathway mechanism governs in situ MgAl_2_O_4_ spinel formation in alumina matrix.
- The optimal kaolin/MgO ratio of 4:1 yields a 17.74 kV/mm characteristic breakdown strength. Excess MgO incorporates into glassy phase, inducing ionic conduction channels.
- A microstructure–defect–breakdown correlation model is established and provides a theoretical basis for optimizing low-cost alumina energy storage ceramics.

**Abstract:**

The escalating demands of pulsed power technology and high-energy-density capacitors necessitate dielectric ceramics with simultaneously enhanced breakdown reliability and microstructural homogeneity. Herein, Al_2_O_3_-MgAl_2_O_4_ composites were fabricated via a solid-state reaction using 80 wt% α-Al_2_O_3_ with kaolin/MgO mass ratios of 7:1, 4:1, and 1:1. XRD presents a dual-pathway spinel formation mechanism. SEM reveals 2–5 μm columnar spinel grains uniformly dispersed at grain boundaries. Porosity decreased from 4.29% to 2.69% while grain size increased from 3.35 to 5.64 μm as MgO content rose. Weibull analysis (n ≥ 10) showed that the 4:1 group achieved optimal dielectric reliability with a characteristic breakdown strength of 17.74 kV/mm and a Weibull modulus of 16.07, representing 8.76% and 20.93% improvements over the 7:1 (16.31 kV/mm, m = 24.58) and 1:1 (14.67 kV/mm, m = 15.37) groups, respectively. The 4:1 composition balanced grain boundary pinning (4.61 μm) and residual porosity (3.59%), maximizing interfacial charge scattering. Excess MgO (1:1) introduced residual Mg^2+^ into the glassy phase, inducing ionic conduction and premature breakdown. This study establishes a quantitative “microstructural composition–defect size distribution–dielectric breakdown” correlation, providing a theoretical foundation for microstructural optimization of low-cost alumina-based energy storage ceramics.

## 1. Introduction

The rapid development of pulsed power technology, high-energy-density capacitors, and advanced power electronic devices has imposed increasingly stringent requirements on dielectric energy storage materials [1,2]. Alumina (Al_2_O_3_) ceramics are widely utilized as substrate and packaging materials owing to their excellent chemical stability, high thermal conductivity, and relatively high dielectric strength. The dielectric breakdown strength (DBS) of Al_2_O_3_-based ceramics is highly sensitive to purity, densification, microstructural homogeneity, and specimen thickness [3]. For fully dense alumina, DBS values can exceed 30 kV/mm under optimized conditions [4]. However, conventional industrial-grade compositions typically exhibit a DBS in the range of 10–20 kV/mm, with higher values achievable through advanced processing routes [5,6]. Nevertheless, the intrinsically low dielectric constant of single-phase alumina (ε_r_ ≈ 8–10) and the extreme sensitivity of its breakdown process to microscopic defects (pores, impurities, and weak grain boundary regions) severely restrict the enhancement of energy storage density (U_e_ = ½ε_0_ε_r_E^2^), which is fundamentally limited by the stochastic dispersion of dielectric breakdown field strength [7,8,9,10,11].

Composite ceramic design, which introduces a second phase to modulate the matrix microstructure, is recognized as an effective strategy to overcome the performance bottlenecks of single-phase materials [10,11,12]. Magnesium aluminate spinel (MgAl_2_O_4_, MA) is particularly advantageous as a secondary phase due to its compatible thermal expansion coefficient with Al_2_O_3_ (αMA ≈ 7.6 × 10^−6^ K^−1^; αAl_2_O_3_ ≈ 8.0 × 10^−6^ K^−1^) [13,14], excellent high-temperature chemical stability, and cubic crystal structure that effectively mitigates stress concentration at grain boundaries. More importantly, the incorporation of the MgAl_2_O_4_ second phase can substantially alter the charge distribution and electric field concentration at grain boundaries, thereby influencing the dielectric breakdown path of the material [15,16].

Grain boundary engineering (GBE) theory posits that the active manipulation of second-phase species, content, and distribution at grain boundaries enables the deliberate intervention of dielectric breakdown processes [17,18,19]. In alumina-based composite systems, the spinel second phase may function as a charge scattering center to dissipate local electric field concentrations; however, it may also form low-dielectric-constant or high-conductivity channels at grain boundaries, potentially inducing premature breakdown [20,21]. Consequently, a theoretical “optimal value” of second-phase content exists, corresponding to a balance between interfacial polarization effects and breakdown reliability [22,23].

Existing studies have predominantly focused on the mechanical properties and high-temperature stability of MgAl_2_O_4_-Al_2_O_3_ composite materials, while systematic investigations into their dielectric breakdown behavior and energy storage characteristics remain insufficient [24,25,26]. In particular, kaolin (primarily Al_2_O_3_·2SiO_2_·2H_2_O), serving as a source of Al_2_O_3_ and SiO_2_ [27], decomposes during thermal treatment to yield reactive Al_2_O_3_ that reacts with externally added MgO. The resulting reaction pathway, and the impact of the excess MgO on breakdown performance, remain mechanistically ambiguous [28].

In this context, the present study employs 80 wt% industrial alumina as the matrix and modulates the kaolin/MgO ratio to investigate the microstructural evolution of the MgAl_2_O_4_ second phase and its quantitative influence on dielectric breakdown strength. The breakdown reliability of different compositions was evaluated using the two-parameter Weibull statistical model. By integrating porosity and grain size data, a quantitative correlation linking microstructural composition, grain boundary architecture, and dielectric breakdown was established, aiming to provide theoretical guidance for the microstructural optimization of Al_2_O_3_-based energy storage ceramics.

## 2. Materials and Methods

### 2.1. Sample Preparation

**Figure 1:**
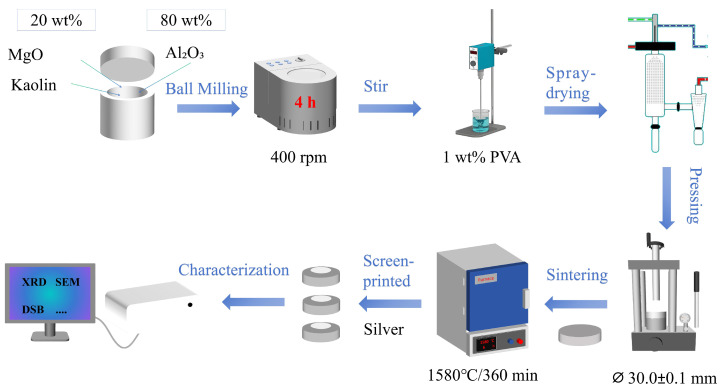
A schematic of the experimental procedure.

**Figure 2:**
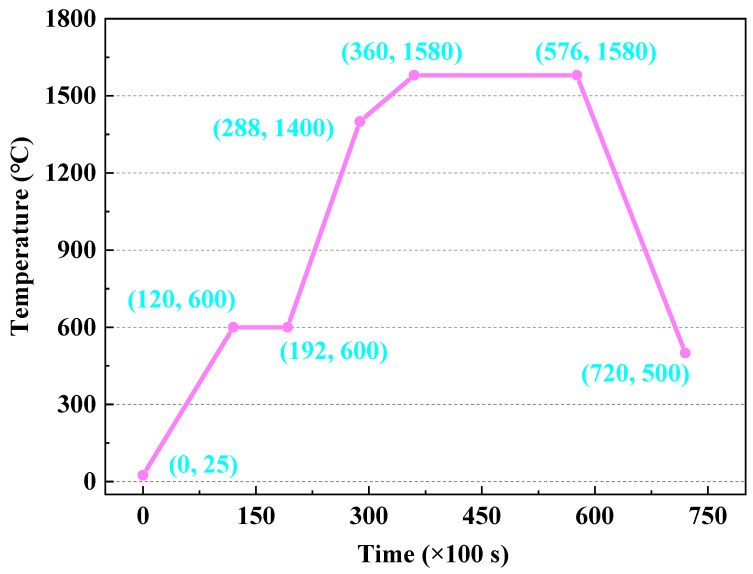
The specific sintering curve. The programmed cooling was controlled from 1580 °C down to 500 °C to protect the furnace heating elements and refractory lining from thermal shock; below 500 °C, the furnace was shut off and samples were cooled to room temperature naturally.

Industrial α-Al_2_O_3_ (purity ≥99%, median particle size 5.13 μm) was used as the matrix raw material. Low-cost kaolin (chemical composition listed in Table 1) served as the reactive aluminum source, and light-burned MgO was employed as the magnesium source. The raw materials were weighed according to the formulations presented in Table 2. The mixed powders were ball-milled with deionized water and milling media in a polyurethane jar at 400 rpm for 6 h. After separating the milling media, 1 wt% polyvinyl alcohol (PVA) binder was added to the slurry, which was subsequently spray-dried to obtain granulated powders. Disk-shaped specimens with a diameter of 30 mm were uniaxially pressed, targeting a sintered thickness of approximately 1.8 mm. A schematic of the experimental procedure is shown in Figure 1. No pre-calcination of kaolin was performed; instead, the dehydroxylation of kaolinite was accommodated during the unified sintering cycle (Figure 2) via a holding state at 600 °C, allowing for gradual release of structural water without cracking or bloating.

The sintering schedule was determined based on the formation kinetics of magnesium aluminate spinel. MgAl_2_O_4_ begins to crystallize above 1100 °C, forms extensively in the 1200–1300 °C range, but requires prolonged holding at approximately 1600 °C for full densification. After optimization of shrinkage and deformation behavior, the optimal sintering temperature was established at 1580 °C. To minimize thermal deformation of the thin (target thickness ~1.8 mm) disks during sintering at 1580 °C, all disks were checked for flatness using a micrometer; specimens with a thickness variation <0.05 mm were accepted, and slightly warped specimens were ground flat before testing. The specific heating profile is shown in Figure 2. To prevent thermal shock damage to the MoSi_2_ heating elements and furnace refractory, the programmed cooling was controlled from 1580 °C to 500 °C. Below 500 °C, the furnace was powered off, allowing the specimens to cool naturally to room temperature inside the chamber—a standard practice that does not induce thermal cracking in alumina-based ceramics given the low thermal stress below this temperature threshold.

### 2.2. Characterization and Testing

Phase composition was analyzed by X-ray diffraction (XRD, SmartLab SE, RIGAKU, Tokyo, Japan) over a 2θ range of 5–90° using Cu Kα radiation. The microstructure was examined by scanning electron microscopy (SEM, Nova NanoSEM 430, Thermo Fisher Scientific, Waltham, MA, USA). For SEM observation, specimens were fractured to expose fresh surfaces, etched with HF, ultrasonically cleaned, and sputter-coated with platinum. Grain size was determined by the linear intercept method (ISO 13383-1:2012) [29]. Porosity was quantified via binary image processing of backscattered electron (BSE) micrographs using ImageJ software (version ij154, National Institutes of Health, Bethesda, MD, USA).

Dielectric breakdown strength testing was performed using a 1.8 mm diameter sphere–sphere electrode system in No. 25 transformer oil, with voltage ramped at 1.0 kV/s under AC conditions at 50 Hz until breakdown (IBVAD-50, Guilin Saimei Testing Technology Co., Ltd., Guilin, China) [30]. At least 10 specimens were tested per group. The breakdown voltage (kV) was recorded for each specimen and divided by its measured thickness (mm) to obtain the dielectric breakdown strength (DBS, kV/mm). The DBS data were statistically processed using Weibull distribution:

(1) $$P\left(E\right)=1-exp\left[-{\left(\frac{E}{E_{0}}\right)}^{m}\right]$$

where E is the measured breakdown strength, E_0_ is the characteristic breakdown strength, and m is the Weibull modulus, which characterizes data dispersion and material reliability. Linearization via double logarithmic transformation yields the following:

(2) $$ln\left[-ln\left(1-P\right)\right]=mln\left(E\right)-mln\left(E_{0}\right)$$

## 3. Results and Discussion

### 3.1. Phase Composition and Second-Phase Formation Mechanism

**Figure 3:**
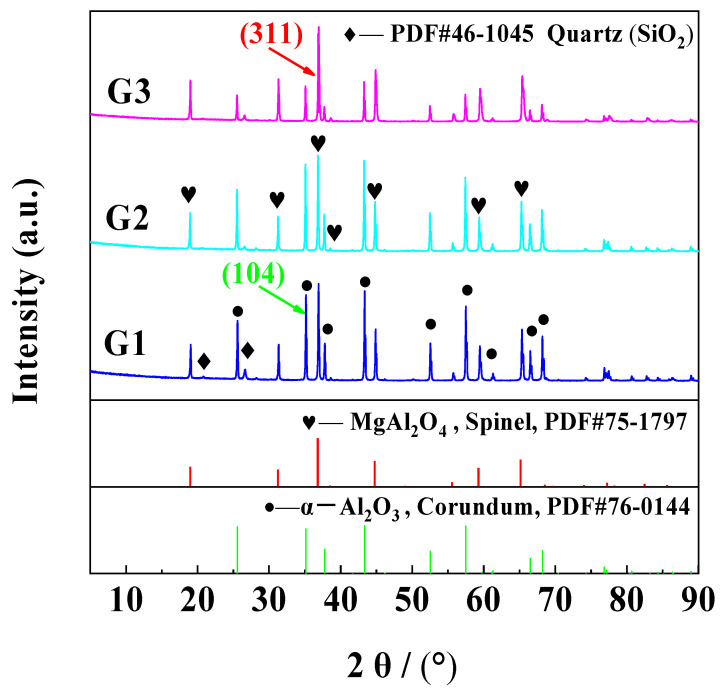
The XRD patterns of the sintered sample.

Figure 3 presents the XRD patterns of the samples. All specimens exhibited diffraction peaks corresponding to corundum-phase α-Al_2_O_3_ (PDF#76-0144) and magnesium aluminate spinel MgAl_2_O_4_ (PDF#75-1797). A trace peak at 2θ ≈ 26.6° is identified as residual quartz (SiO_2_, PDF#46-1045), originating from the incomplete reaction of the minor quartz impurity in raw kaolin; its negligible intensity indicates a content far below the threshold required to affect the spinel formation or dielectric properties, and thus it is not discussed further. As the kaolin/MgO ratio decreased from 7 (G1) to 1 (G3), the intensity of the MgAl_2_O_4_ (311) reflection (2θ ≈ 36.8°) increased significantly from G1 to G3, consistent with the enhanced spinel formation at higher MgO loadings. Concurrently, the relative intensity of the α-Al_2_O_3_ (104) peak (2θ ≈ 35.1°) decreased correspondingly, reflecting the dilution of the alumina matrix by the in situ formed spinel phase.

The formation of MgAl_2_O_4_ in this system is governed by a dual-pathway reaction mechanism. Pathway A (high reactivity): During thermal treatment, kaolinite undergoes dehydroxylation and transforms into a metakaolin or Si-Al-O spinel-like precursor (a highly reactive Al-rich intermediate, not a Mg-bearing silicate spinel), which serves as a highly reactive Al source that reacts with MgO above 1100 °C to form MgAl_2_O_4_. Pathway B (low reactivity): When MgO is in excess, the Al source from kaolin is insufficient to consume all MgO, and the residual MgO reacts with the raw α-Al_2_O_3_. However, owing to the intact lattice and low reactivity of industrial α-Al_2_O_3_, this pathway is kinetically sluggish. Consequently, as the kaolin/MgO ratio decreases, a certain proportion of MgO fails to enter the spinel lattice.

Quantitative XRD analysis (estimated by the reference intensity ratio, RIR, method) revealed that the increase in the MgAl_2_O_4_/Al_2_O_3_ diffraction intensity ratio from G1 to G3 was not proportional to the MgO increment (6.2 wt% (G1), 8.4 wt% (G2), 12.7 wt% (G3)). When the MgO content increased from 2.5 wt% (G1) to 10 wt% (G3), the relative MgAl_2_O_4_ content did not increase as expected. This confirms the inefficiency of Pathway B: excess MgO does not effectively convert to crystalline spinel but rather remains in an amorphous state within the high-temperature liquid phase.

### 3.2. Microstructural Characteristics

**Figure 4:**
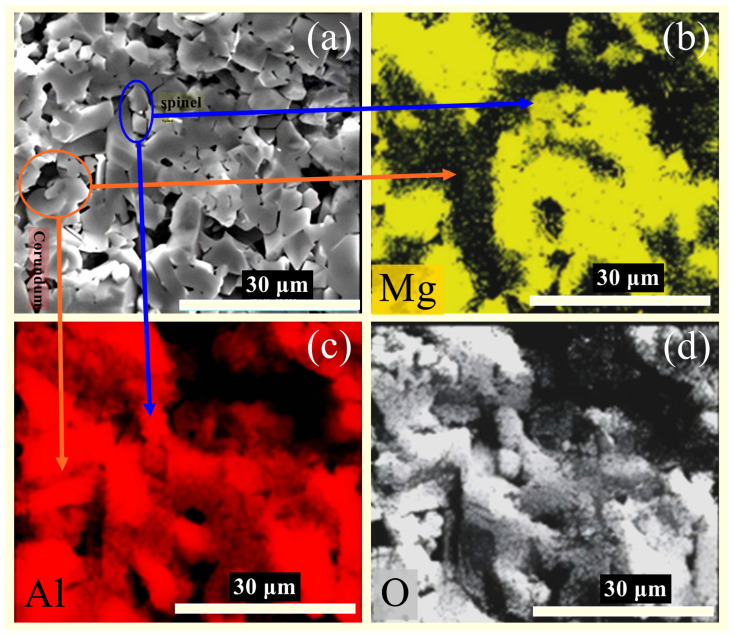
High-magnification elemental mapping of sample G2. All images are captured at the same magnification with a unified scale bar of 30 μm for direct visual comparison: (**a**) secondary electron (SE) image; (**b**) Mg map; (**c**) Al map; (**d**) O map.

High-magnification elemental mapping of sample G2 (Figure 4) confirmed that the columnar microcrystals were enriched in Mg and Al, which, in combination with XRD results, identifies them as magnesium aluminate spinel. Notably, the spinel phase was uniformly dispersed within the liquid-phase regions surrounding the alumina grains, forming a distinctive grain boundary pinning network.

**Figure 5:**
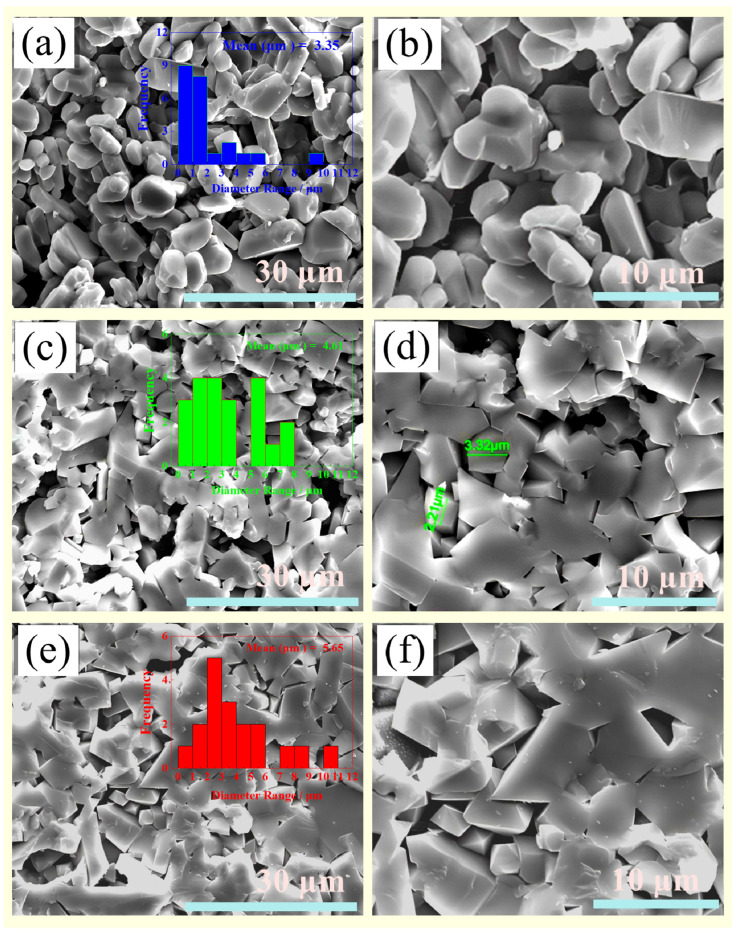
SEM images of sintered samples at different kaolin/MgO ratios ((**a**,**b**), G1; (**c**,**d**), G2; (**e**,**f**), G3).

SEM images (Figure 5) revealed a typical multiphase microstructure in all three groups: the matrix consisted of elongated α-Al_2_O_3_ primary grains (length ~10–20 μm), while smaller short-columnar MgAl_2_O_4_ crystals (size ~2–5 μm) were distributed around the elongated grains. In the G1 sample (7:1 ratio), the grain size was the smallest (average ~3.35 μm) with a moderate interfacial density. As the MgO content increased and the kaolin content decreased, the glassy-phase fraction increased in the microstructure. This suggests that excess MgO reacts with SiO_2_ to promote the formation of low-melting-point liquid phases. An appropriate amount of high-temperature liquid phase facilitates atomic migration, benefiting the nucleation and growth of spinel; however, excessive liquid phase forms continuous, weak glassy films at grain boundaries.

**Figure 6:**
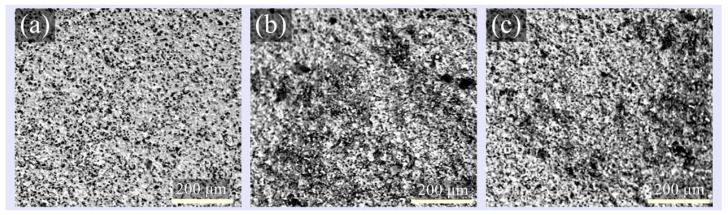
Backscattered electron (BSE) images of sintered samples at different kaolin/MgO ratios ((**a**), G1; (**b**), G2; (**c**), G3).

BSE image analysis (binary processing via ImageJ, Figure 6 and Table 3) showed that as the kaolin/MgO mass ratio decreased, the porosity (Table 3) decreased monotonically (G1: 4.29% → G3: 2.69%), attributable to liquid-phase-promoted densification. However, the average grain size increased concurrently (G1: 3.35 μm → G3: 5.64 μm), indicating that a greater amount of high-temperature liquid phase enhanced grain growth.

### 3.3. Weibull Reliability Analysis of Dielectric Breakdown Performance

**Figure 7:**
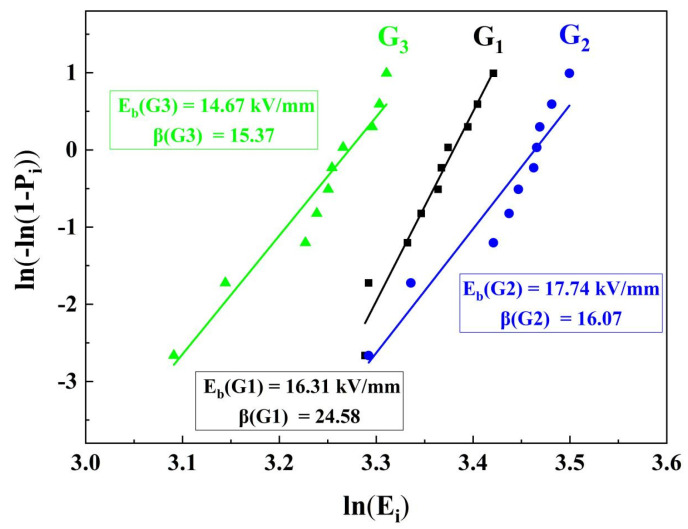
Weibull probability plots for the sintered samples prepared with different kaolin/MgO ratios.

The Weibull statistical analysis results are presented in Figure 7 and Table 4. To contextualize the performance of the present composite system, our results are compared with representative alumina-based ceramics reported in the literature. Touzin et al. reported an alumina ceramic containing ~2 wt% spinel with a DBS of 15.1 ± 0.8 kV/mm (t = 3 mm) [30]. Xu et al., our group’s prior work on fractal dendritic Ca_9_Al(PO_4_)_7_-reinforced alumina (70 wt% Al_2_O_3_), achieved a DBS of 16.1 kV/mm [10]. Haddour et al. reported conventional alumina with DBS values of 13.0~14.9 kV/mm [31]. Against these benchmarks, the G2 sample in this study (kaolin/MgO = 4:1) attains a characteristic breakdown strength of 17.74 kV/mm with m = 16.07, representing a competitive improvement attributable to the optimized in situ columnar spinel distribution and grain boundary pinning architecture.

The characteristic breakdown strength initially increased and subsequently decreased with decreasing kaolin/MgO ratio. Synthesizing the above analyses, we propose a “microstructural composition—defect size distribution—dielectric breakdown” correlation model for the Al_2_O_3_-MgAl_2_O_4_ composite ceramics. This model encompasses three competing mechanisms.

Mechanism I (Enhancement Effect). The G2 group achieved a characteristic breakdown strength of 17.74 kV/mm, representing an 8.76% improvement over G1 (16.31 kV/mm) and a 20.93% improvement over G3 (14.67 kV/mm). At a kaolin/MgO ratio of 4:1, the reactive Al_2_O_3_ derived from kaolin decomposition reacts completely with MgO, generating an optimal amount of MgAl_2_O_4_ second phase. The moderate spinel content refines the grains via grain boundary pinning, increases the density of heterogeneous interfaces, and homogenizes the local electric field distribution, thereby enhancing the breakdown strength. This mechanism dominates in the G2 group.

Mechanism II (Homogenization Effect). The G1 group exhibited a lower characteristic breakdown strength than G2. This performance limitation does not stem from excessive spinel content, but rather from insufficient MgO loading, which produces an inadequate amount of spinel second phase and a correspondingly low grain boundary interfacial density. The limited charge scattering and electric field dispersion effects are therefore restricted. Simultaneously, the finer crystalline structure in G1 (e.g., smaller grains) confers a highly uniform defect size distribution, reducing the statistical dispersion of breakdown data (high m value) but constraining the intrinsic strength enhancement.

Mechanism III (Degradation Effect). For the G3 sample, excess MgO exhausts the Al_2_O_3_ supplied by kaolin, and a substantial quantity of unreacted MgO enters the high-temperature glassy network. Under AC electric field conditions, Mg^2+^ and other alkaline-earth metal ions (naturally present as minor impurities such as CaO and Fe_2_O_3_ in kaolin) exhibit high mobility within the glassy phase, forming ionic conduction channels. Furthermore, under extensive high-temperature liquid-phase conditions, a larger volume fraction of spinel phase (MgAl_2_O_4_, ε_MA_ ≈ 8.0) is formed [20,32], effectively diluting the higher-permittivity alumina matrix (ε_Al2O3_ ≈ 8.5–9.0) and reducing the overall effective breakdown field. Additionally, the presence of excessive glassy phase diminishes the mechanical strength at grain boundaries, making intergranular breakdown more susceptible to initiation under coupled electrical–mechanical stress. Consequently, the dielectric breakdown performance of this group deteriorates sharply.

The competition among these three mechanisms governs the non-monotonic variation in breakdown strength and Weibull modulus. For the 80 wt% Al_2_O_3_-based composite system investigated herein, the optimal value corresponds to a kaolin/MgO ratio of approximately 4:1, at which the second-phase content is moderate, the porosity is ~3.6%, the average grain size is ~4.5–5.0 μm, and the characteristic breakdown strength reaches *E_0_* = 17.74 kV/mm.

## 4. Conclusions

This study elucidates the quantitative regulatory effects of MgAl_2_O_4_ second-phase formation on microstructure and dielectric breakdown reliability in 80 wt% Al_2_O_3_-based composites. A quantitative “microstructural composition–defect size distribution–dielectric breakdown” correlation for Al_2_O_3_-MgAl_2_O_4_ composites fabricated via in situ solid-state reaction is established. The optimal kaolin/MgO ratio of 4:1 yields a characteristic breakdown strength of 17.74 kV/mm with a Weibull modulus of 16.07, representing improvements of 8.76% and 20.93% over the 7:1 (16.31 kV/mm, m = 24.58) and 1:1 (14.67 kV/mm, m = 15.37) compositions, respectively. This enhancement arises from balanced grain boundary pinning by columnar spinel (2–5 μm) and moderated residual porosity (~3.6%), which maximize interfacial charge scattering. Excess MgO (1:1 ratio) degrades performance by introducing ionic conduction channels via residual Mg^2+^ incorporation into the glassy phase, whereas insufficient MgO (7:1 ratio) limits strength due to low interfacial density despite high statistical reliability (m = 24.58). The analytical framework—encompassing the dual-pathway spinel formation mechanism and defect distribution homogeneity—offers a transferable theoretical basis for microstructural optimization of low-cost alumina-based energy storage ceramics and related oxide composite systems.

## Figures and Tables

**Table 1:** Chemical composition of kaolin (wt%).

Sample	SiO_2_	Al_2_O_3_	Fe_2_O_3_	K_2_O	Others	L.O.I.
Kaolin	50.87	37.96	0.57	0.37	0.02	10.21

**Table 2:** Experimental formulation (wt%).

Group	Al_2_O_3_	MgO	Kaolin	Kaolin/MgO (Mass Ratio)
G1	80	2.5	17.5	7:1
G2	80	4.0	16.0	4:1
G3	80	10.0	10.0	1:1

**Table 3:** Microstructural parameters of the samples.

Group	Kaolin/MgO	Average Grain Size (μm)	Porosity (%)
G1	7	3.35	4.29
G2	4	4.61	3.59
G3	1	5.64	2.69

**Table 4:** Two-parameter Weibull statistical results.

Group	Characteristic Breakdown Strength (kV/mm)	Weibull Modulus (m)
G1	16.31	24.58
G2	17.74	16.07
G3	14.67	15.37

## Data Availability

The original contributions presented in this study are included in the article. Further inquiries can be directed to the corresponding authors.
